# Morbidity and mortality due to malaria in Est Mono district, Togo, from 2005 to 2010: a times series analysis

**DOI:** 10.1186/1475-2875-11-389

**Published:** 2012-11-23

**Authors:** Essoya D Landoh, Potougnima Tchamdja, Bayaki Saka, Khin S Tint, Sheba N Gitta, Peter Wasswa, de Jager Christiaan

**Affiliations:** 1 Division de l’Epidémiologie, Ministère de la Santé du Togo, BP: 1396, Lomé, Togo; 2 UP Centre for Sustainable Malaria Control, School of Health Systems & Public Health, University of Pretoria, Pretoria, South Africa; 3 FacultéMixte de Médecineet de Pharmacie, Université de Lomé, Lomé, Togo; 4 South African Field Epidemiology and Laboratory Training Programme (SAFELTP), National Institute for Communicable Diseases, Johannesburg, South Africa; 5 African Field Epidemiology Network (AFENET), Kampala, Uganda

**Keywords:** Malaria, Mortality, Morbidity, ACT, Rainfall, Time series analysis, Togo

## Abstract

**Background:**

In 2004, Togo adopted a regional strategy for malaria control that made use of insecticide-treated nets (ITNs), followed by the use of rapid diagnostic tests (RDTs), artemisinin-based combination therapy (ACT). Community health workers (CHWs) became involved in 2007. In 2010, the impact of the implementation of these new malaria control strategies had not yet been evaluated. This study sought to assess the trends of malaria incidence and mortality due to malaria in Est Mono district from 2005 to 2010.

**Methods:**

Secondary data on confirmed and suspected malaria cases reported by health facilities from 2005 to 2010 were obtained from the district health information system. Rainfall and temperature data were provided by the national Department of Meteorology. Chi square test or independent student’s t-test were used to compare trends of variables at a 95% confidence interval. An interrupted time series analysis was performed to assess the effect of meteorological factors and the use of ACT and CHWs on morbidity and mortality due to malaria.

**Results:**

From January 2005 to December 2010, 114,654 malaria cases (annual mean 19,109 ± 6,622) were reported with an increase of all malaria cases from 10,299 in 2005 to 26,678 cases in 2010 (p<0.001). Of the 114,654 malaria cases 52,539 (45.8%) were confirmed cases. The prevalence of confirmed malaria cases increased from 23.1 per 1,000 in 2005 to 257.5 per 1,000 population in 2010 (p <0.001). The mortality rate decreased from 7.2 per 10,000 in 2005 to 3.6 per 10,000 in 2010 (p <0.001), with a significant reduction of 43.9% of annual number of death due to malaria. Rainfall (β-coefficient = 1.6; p = 0.05) and number of CHWs trained (β-coefficient = 6.8; p = 0.002) were found to be positively correlated with malaria prevalence.

**Conclusion:**

This study showed an increase of malaria prevalence despite the implementation of the use of ACT and CHW strategies. Multicentre data analysis over longer periods should be carried out in similar settings to assess the impact of malaria control strategies on the burden of the disease. Integrated malaria vector control management should be implemented in Togo to reduce malaria transmission.

## Background

Malaria remains a major public health concern. According to the World Malaria Report 2010, the global prevalence of the disease was estimated at 225 million cases and 781,000 deaths in 2009 [1]. About 80% of these cases occur in sub-Saharan Africa, with rural areas that have poor access to health services being most affected [2,3]. In Togo, malaria is a priority disease because of the high morbidity and mortality associated with it. The disease accounted for 39% of out-patient consultations, 21% of hospital admissions and 4.9% of case fatalities in 2007 [4].

Malaria control strategies primarily comprise prevention through vector control, such as indoor residual spraying (IRS) or the use of personal preventive methods such as bed nets and mosquito repellents followed by chemoprophylaxis. Control also takes the form of effective case management and medication. The implementation of these strategies has been supported by donor funding and the commitment of governments and partners from many countries [5]. In many developing countries there is variable success in vector control using insecticide-treated bed nets (ITNs) and IRS with dichlorodiphenyltrichloroethane (DDT) or other agents [6,7].

Since 2004, the Togolese government has adopted a regional strategy of malaria control through the use of ITNs, the use of artemisinin-based combination therapy (ACT) as well as community involvement [4]. Malaria control strategies in Est Mono district include promotion of the use of ITNs in the community, which is one of the main technical approaches of the Roll Back Malaria campaign (RBM), implemented in late 2004 in Togo. In 2007, the country started the implementation of artemether–lumefantrine as first-line treatment for uncomplicated malaria in all public health facilities and by community health workers (CHWs) because rapid diagnostic tests (RDTs) to confirm malaria could be carried out even in health facilities that did not have the laboratory capacity. CHWs were trained to promote health, treat uncomplicated malaria cases within their communities and report data to their respective health care centres. These strategies aimed at increasing the accessibility of health care to isolated populations and those populations living beyond five kilometres of health facilities and to reduce morbidity and mortality due to malaria by 50% in 2012 from 2005 levels [4].

After five years, the impact of the implementation of the new malaria control strategies on the malaria burden in Est Mono district remained unknown. The objective of this study was to assess the trends of malaria prevalence and specific mortality due to malaria in Est Mono district from 2005 to 2010.

## Methods

### Design of the study

The study took the form of a retrospective secondary data analysis of malaria trends from 2005 to 2010. The district collects health information routinely from health facilities and compiles the data on a monthly basis. The completeness of data in the district was 97% during the study period [8].

### Setting

Est Mono district covers an area of 2,474 km^2^ with a population of 89,060 inhabitants and a population growth rate of 1.03% in 2010 [8]. Est Mono is one of the nine health districts in the Plateaux region, which is one of Togo’s six regions. The district is situated in central Togo and has 17 health facilities run by nurses, auxiliary nurses, or non-qualified nurses. Each health unit has CHWs trained in health promotion and the management of uncomplicated malaria cases within their communities.

Est Mono district [8] and the other central and southern parts of Togo have two rainy seasons, the first starting in April and ending in July and the second starting in September and ending in late November. The district receives on average 949 mm (±37.4) of precipitation annually or 79 mm (±3.1) each month. The climate in Togo is generally tropical, with average temperatures ranging from 27.5°C (81.5°F) on the coast to about 30°C (86°F) in the north. Malaria transmission is seasonal, with peaks related to rainfall.

### Data collection

Secondary aggregated data on confirmed and suspected malaria cases reported by health facilities from 2005 to 2010 were obtained from the Malaria Control Programme of the district health information system. The population estimates were obtained from the district to calculate prevalence and specific mortality rate due to malaria [8]. Information on the number of CHWs trained each year was gained from the district register. Data on ACT were abstracted from the monthly health facility reports sent to the district. Rainfall and temperature data from 2005 to 2010 were provided by the Department of Meteorology in Togo.

### Data analysis

Suspected malaria cases were defined as any person presenting with fever (>38°C) for less than two days or having a history of fever associated with symptoms such as nausea, vomiting and diarrhoea, headache, back pain, chills, myalgia, where other infectious diseases have been excluded and a treatment using anti-malarial drugs was successful. Confirmed cases were persons with suspected malaria who had been shown to have malaria parasites by using a parasitological test such as microscopy (using a blood smear) or RDT. RDT was used in the health facilities where there was no laboratory and also by CHWs, according to the national malaria control guidelines [4].

Individual-level data were not available and consequently data on age or sex distribution were not provided. However, aggregated data on targeted groups (under five years old, more than five years old and pregnant women) were available. Malaria prevalence and specific mortality rates were calculated using the mid-year population, which was estimated from population data based on the 1980 census and assuming a 1.03% annual population growth each year. The prevalence of malaria per 1,000 people was calculated per year.

The malaria mortality rate was defined as the number of deaths among persons identified as suspected or confirmed malaria cases divided by the mid-year population of the district, while prevalence was defined as the number of reported malaria cases divided by the mid-year population.

The Chi square test was used to compare trends in malaria prevalence in the district and the mortality rate over the six years at a 95% confidence interval. The Student t-test was performed to compare the rainfall means and mean temperatures. An interrupted time series analysis was performed to assess the effect of rainfall, temperature [9,10], and the use of ACT and CHWs on the transmission of malaria by fitting the ARIMA model (Autoregressive Integrated Moving Average) to calculate trends using the moving average of monthly suspected and confirmed malaria cases. A segmented or piece-wise regression [11,12] was performed for annual reported malaria cases considering the joint point to be 2007 when ACT and CHW strategies were initiated. Data on the number of malaria cases were only available from 2005 onwards and this limited the possibility to use a sufficiently long time series analysis on an annual basis. Analyses were performed using Stata version 11.0.

### Ethical issues

This study was approved by the Faculty of Health Sciences Ethics Committee at the University of Pretoria, South Africa (Ref No. 79/2010: 04/06/2010) and the Ministry of Health of Togo (Ref No. 1420/2009/MS/DGS/DSSP/DEPI).

## Results

### Malaria cases reported

All reported outpatient and reported malaria cases are shown in Table 1. During the study period, the proportion of the population using the health care service in Est Mono district increased from 29% (22,852/79,000) in 2005 to 62% (55,603/89,060) in 2010 (χ^2^ = 5742 p < 0.001) (Table 1).

**Table 1:** Trends in malaria cases reported in Est Mono district from 2005 to 2010

**Years**	**Population of district**	**Out patient all causes**	**Total reported malaria cases N (%)**	**Reported malaria cases**		**Prevalence of malaria all cases (1/1,000)**	**Prevalence confirmed cases (1/1,000)**	**No. of reported death due to malaria**	**Specific mortality rate (1/10,000)**
				**Suspected cases**	**confirmed cases**				
**2005**	79,000	22,852	10,299 (45.1)	8,472	1,827	130.4	23.1	57	**7.2**
**2006**	81,000	25,354	12,843 (50.7)	11,961	882	158.6	10.9	57	**7.0**
**2007**	82,673	31,819	17,776 (55.9)	12,777	4,999	215.0	60.5	53	**6.4**
**2008**	84,931	39,834	22,068 (55.4)	12,019	10,049	259.8	118.3	58	**6.8**
**2009**	86,980	46,876	24,990 (53.3)	13,143	11,847	287.3	136.2	24	**2.8**
**2010**	89,060	55,603	26,678 (48.0)	3,743	22,935	299.6	257.5	32	**3.6**

**Figure 1:**
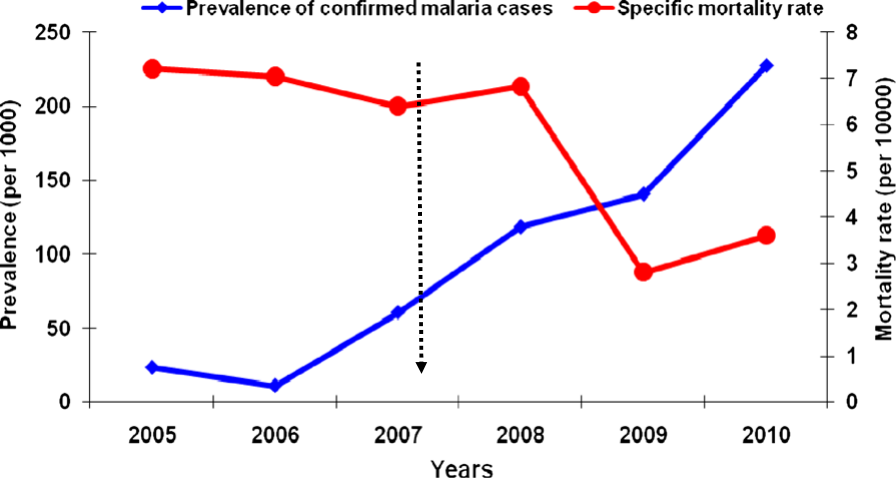
Change in malaria prevalence and mortality from 2005 to 2010 in Est Mono district before and after implementation of ACT and CHW involvement strategies.

From 2005 to 2010 114,654 malaria cases (mean 19109 ± 6622) were reported in Est Mono district with an overall increase of total malaria cases by 159.03% from 10,299 cases in 2005 to 26,678 cases in 2010 (χ^2^ = 702.8; p <0.001) (Figure 1). Of the 114,654 malaria cases, 52,539 (45.8%) were confirmed cases (Table 1).

The annual prevalence of confirmed malaria cases increased steeply from 23.1 per 1,000 population in 2005 to 257.5 per 1,000 in 2010 (χ^2^ = 7000.7; p <0.001), indicating a significant increase over time (Table 1). Furthermore, the proportion of confirmed cases increased from 17.7% (1,827/10,299) in 2005 to 85.9% (22,935/26,678) in 2010. A total of 281 deaths were reported in the district between 2005 and 2009. The mortality rate decreased from 7.2 per 10,000 in 2005 to 3.6 per 10,000 in 2010 (χ^2^ = 15.9; p <0.001), with a significant reduction of 43.9% in the annual number of death due to malaria between 2005 and 2010 (Figure 1).

From 2005 to 2010, the number of suspected and confirmed malaria cases reported was higher among children under five years (Table 2). The proportion of confirmed malaria cases was 46.6% (24,473/52,539) among the population over five years of age, 45.6% (23,979/52,539) among the population below five years, and 7.8% (4,087/52,539) in pregnant women (Table 3).

**Table 2:** Trends of suspected and confirmed malaria cases among specific target groups in Est Mono district from 2005 to 2010

**Years**				**Children < 5 year**		**Patients** ≥**5 years**		**Pregnant Women**		**Total cases**
	**N**	**Percent (%)**	**N**	**Percent (%)**	**N**	**Percent (%)**				
2005	4974	48.3	5079	49.3	246	2.4	10299
2006	5571	43.4	6609	51.5	663	5.2	12843
2007	8358	47.0	8402	47.3	1016	5.7	17776
2008	11285	51.1	9364	42.4	1419	6.4	22068
2009	11624	46.5	11842	47.4	1524	6.1	24990
2010	14418	54.0	10320	38.7	1940	7.3	26678
Total	56230	49.0	51616	45.0	6808	5.9	114654

**Table 3:** Malaria cases among specific target groups in Est Mono district from 2005 to 2010

**Target population group**	**Reported cases (Suspected and confirmed)**		**Confirmed cases**	
	**N**	**%**	**N**	**%**
< 5 years	56,230	49.1	23,979	45.6
≥ 5 years	51,616	46.0	24,473	46.6
Pregnant women	6,808	5.9	4,087	7.8
**Total**	**114,654**	**100.0**	**52,539**	**100.0**

### Availability of ACT and CHW

The number of days per year where there was a shortage of ACT in Est Mono district decreased from 160 in 2007 to 23 in 2010. A total of 220 CHWs were trained in 2007 compared to 310 CHWs trained annually between 2008 and 2010 (Table 4).

**Table 4:** Number of personnel trained in malaria case management in Est Mono district from 2005 to 2010

**Years**	**No. of health workers trained**	**No. of CHW trained**	**No. of lab persons trained**
2005	**0**	**0**	**0**
2006	**0**	**0**	**0**
2007	50	220	3
2008	34	310	0
2009	34	310	2
2010	34	310	2
**Total**	**152**	**1150**	**7**

### Seasonality of malaria

**Figure 2:**
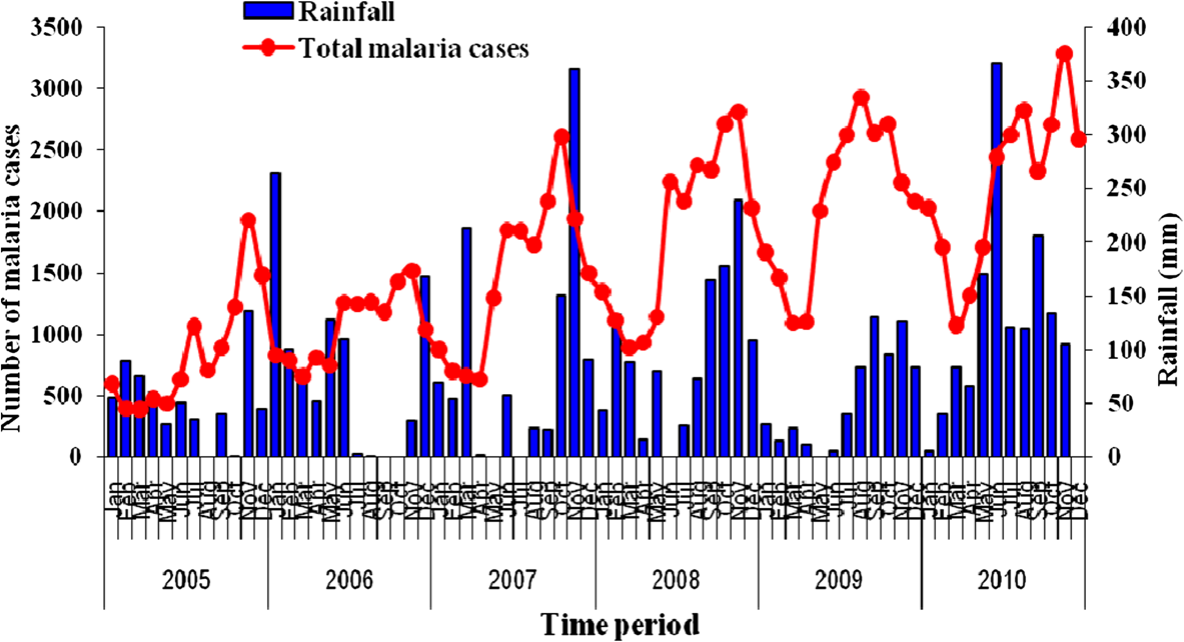
Relationship between monthly malaria transmission and rainfall in Est Mono district from 2005 to 2010.

The level of annual rainfall was similar from 2005 to 2010. The overall median rainfall was 63.1 mm (IQR = 25.9 – 120.5). There was no difference between the mean annual rainfall before 2007 and after 2007 (t = −1.34; p = 0.18). The mean annual maximum temperature was 31.8°c (± 1.7) and the mean minimum annual temperature was 24.8 (± 1.1). Malaria transmission was seasonal in Est Mono district, with each year an increase in overall cases between September and March. The number of reported malaria cases was higher from 2008 to 2010 (Figure 2).

In time series analysis, the rainfall (β coefficient = 1.62; p = 0.05) and the number of CHWs trained (β coefficient = 6.7; p = 0.002) were positively correlated with the number of malaria cases reported (Table 5). There was no correlation between the number of CHWs trained and the mortality due to malaria.

**Table 5:** Time series analysis (ARIMA): Effect of availability of ACT and number of CHWs trained on morbidity and mortality due to malaria in Est Mono district

**Variables**	**Coefficient**	**p-value**	**95% CI**
Effect on morbidity (number of suspected and confirmed malaria cases)			
**Rainfall**	1.62	0.054	−0.028 – 3.258
**Average temperature**	−219.67	0.000	−318.523 – -120.827
**Number of CHWs trained**	6.779	0.002	2.486 – 11.073
**Use of ACT**	−982.90	0.129	−2253.491 – 287.687
**Constant**	7136.4	0.000	4371.517 – 9901.284
Effect on mortality (number of death attributed to malaria)			
**Rainfall**	−0.0008	0.841	−0.009 – 0.007
**Average temperature**	−1.107	0.000	−1.610 – -0.604
**Number of CHWs trained**	−0.012	0.178	−0.031 – 0.006
**Use of ACT**	2.748	0.305	−2.508 – 8.005
**Constant**	35.955	0.000	22.259 – 49.651

## Discussion

The data presented here demonstrate that reported malaria cases remain high in Togo. Indeed this study found an overall increase of malaria cases in Est Mono district of 159% in 2010 from the 2005 level. The proportion of confirmed malaria cases also increased from 17.7% in 2005 to 85.9% in 2010. The government targeted a reduction in morbidity and mortality due to malaria by 50% in 2012 [4]. The national health information reports show an increase of malaria incidence from 80 per 1,000 in 2005 to 160 per 1,000 population in 2010 [13]. Several explanations exist for these observations. With the implementation of the new scale-up policy, there has been increased access to affordable interventions for malaria care even to populations that live more than five kilometres from a health centre. Indeed, the cost of ACT in Togo is relatively low (USD2) for a treatment course compared to the average cost of a case treatment (USD10) before 2007 in the public health centres of the district [8]. This is supported by the increase of the total number of health care visit in Est Mono district during the study period. In Kenya, it was shown that the rate of health care use increased when free high-quality care was offered [14].

The implementation of the new policy of malaria control in Togo took into account the enhancement of health care [4]. These findings suggest that several factors had impacted on the number of malaria cases reported throughout the six years – including the training of health personnel in malaria case management; the introduction of a new treatment at lower cost; the involvement of the community through the CHWs; improvement in the monitoring and reporting system and the use of new RDTs that are simple and quick to perform and show high sensitivity. The findings are consistent with those of Nyarango *et al.* from an Eritrean study, where it was reported that the incidence of malaria was influenced by many factors. These factors included the access to diagnosis and treatment, risk perceptions of the net users, the training of health workers, as well as the involvement of community health agents, who increased the proportion of malaria cases treated in the community from 50% to 78% [6].

A study conducted in Uganda in five selected hospitals over a longer time period (1999 to 2009) reported a significant increase in paediatric malaria admission [15]. The reasons given were the abolition of user fees in the hospitals, low intervention coverage and the high malaria transmission. In Malawi, there was no decline in annual malaria prevalence among children from 2001 to 2010 because of the disparity in treated bed coverage and the low coverage of IRS [16].

In contrast, a decline in malaria incidence has been reported in many countries [6,9,14,17-21] but in most of these countries the interventions implemented included IRS and the involvement of CHWs. Similarly, in a much larger study in South Africa, where IRS was implemented and use of ACT was introduced sequentially, a reduction in the incidence of malaria was observed after implementing the use of ACT [22]. In Togo, IRS is an intervention included in the strategic plan [13] but it has not been implemented yet. ITNs remain widely used for malaria control in Togo. Nyarango et al. [6] and Sharp et al. [23] state that ITNs remain the intervention of choice for prevention of morbidity and mortality from malaria in areas of high transmission. But the preventive effect of ITNs depends on their insecticidal properties and physical integrity [24]. In a recent study conducted in Ethiopia the combination of ITNs and IRS was found to be more effective as a control measure [25].

The results of the Togo study suggest that strengthening of the malaria reporting system by the control programme could have increased the number of reported malaria cases in Est Mono. Malaria cases could have been under-reported before the introduction of the new malaria control policy, which included the enhancement of case reporting.

In this study area, there was a decrease in mortality rate from 7.2 per 10,000 in 2005 to 3.6 per 10,000 in 2010 and an increase of laboratory-confirmed cases. These findings are consistent with national data [13]. The steady decline in mortality due to malaria observed in this study has met the national target of reducing mortality by 50% by 2012. The early clinical diagnosis with laboratory confirmation and appropriate treatment using ACT as recommended by the national guidelines are the main factors that could have contributed to the decline in mortality due to malaria in Est Mono district. It is well known that the early clinical diagnosis and laboratory confirmation are essential aspects of effective care and effective case management of malaria [6].

A study conducted in the province of KwaZulu-Natal in South Africa showed that the use of ACT decreased by 97% mortality due to malaria [22]. Similarly, in Zanzibar the decline by 52% in mortality due to malaria among children under five years old was observed after deployment of ACT [21]. The availability of ACT in Togo and personnel training in malaria case management should be reinforced.

The rainfall was correlated with the prevalence of malaria in the Togo study. The findings are consistent with findings elsewhere [6,9,10,26] and confirm the role played by climatic and seasonal factors in the malaria burden. However, the pattern of rainfall did not change markedly over the six years of the study. The time series analysis shows also that the increase in reported malaria cases could be attributed to the role played by the CHWs trained in malaria case management.

### Limitations

Secondary data were analysed in this study. This could have affected the data quality related to precision and the completeness during the data reporting. The study period of six years was short compared to the period used in other studies and could have affected the power of the ARIMA model. However, previous similar studies using five-year data have provided valid results [6]. Some other factors such as migration, socioeconomic conditions, level of knowledge, community attitudes to and practices of malaria control may have influenced the changes in the burden of malaria that were not taken into account. However, the results of this study are likely to be generalizable to other districts of Togo, since health service delivery and the period of malaria control interventions are similar in all districts. The trends of malaria prevalence and mortality are likely to be representative of those for the entire country.

## Conclusion

This study shows an increase of reported malaria cases and a reduction in mortality due to malaria in Est Mono district with the introduction of ITNs, ACT and the involvement of CHWs. Multicentre data analysis over longer periods should be carried out in West African countries where the transmission of malaria is still high to find out the impact of malaria control strategies on the burden of the disease. The introduction of IRS and other integrated vector control management strategies in Togo needs to be considered for reducing malaria transmission in order to meet the Abuja Declaration goals, which are only three years left from deadline.

## Abbreviations

ACT: Artemisinin-based combination therapy; ARIMA: Autoregressive Integrated Moving Average; CHW: Community Health Worker; RBM: Roll Back Malaria; ITN: Insecticide-treated net; IRS: Indoor residual spraying; DDT: Dichlorodiphenyltrichloroethane; RDT: Rapid diagnostic test.

## Competing interests

Authors declare that there is no conflict of interest.

## Authors’ contributions

DEL participated to the study design, undertook the field study, conducted the data collection, analysis and interpretation, and wrote the manuscript. PT was involved in the study design, supervised data collection and participated in data analysis. BS was involved in the data collection, analysis and interpretation and revised the manuscript. KST was involved in the study design, data analysis and interpretation and revised the manuscript. SG and PW participated in data analysis and interpretation of results, in writing and reviewing the manuscript and provided comments on the manuscript. C de Jager was responsible for the conception of the study and its overall scientific management, for analysis and interpretation, and the preparation of the final manuscript. All the authors have read and approved the final manuscript to be submitted for publication.
